# Lack of compensation for COVID-19-related overtime work and its association with burnout among EMS providers in Korea

**DOI:** 10.4178/epih.e2023058

**Published:** 2023-06-15

**Authors:** Ji-Hwan Kim, Jaehong Yoon, Soo Jin Kim, Ja Young Kim, Jinwook Bahk, Seung-Sup Kim

**Affiliations:** 1 Department of Environmental Health Sciences, Graduate School of Public Health, Seoul National University, Seoul, Korea; 2 Department of Rehabilitation Medicine, Seoul National University Hospital, Seoul, Korea; 3 National Traffic Injury Rehabilitation Research Institute, National Traffic Injury Rehabilitation Hospital, Yangpyeong, Korea; 4 Fire Science Research Center, Seoul Metropolitan Fire Service Academy, Seoul, Korea; 5 Department of Public Health, Keimyung University, Daegu, Korea; 6 Department of Social and Behavioral Sciences, Harvard T. H. Chan School of Public Health, Boston, MA, USA

**Keywords:** Emotional exhaustion, Firefighter, Healthcare worker, Organizational practice, Overwork, Paramedic

## Abstract

**OBJECTIVES:**

This study examined the association between lack of compensation for COVID-19-related overtime work (LCCOW) and burnout among emergency medical service (EMS) providers in Seoul, Korea.

**METHODS:**

We conducted a cross-sectional survey of 693 EMS providers in Seoul, Korea. Participants were classified into 3 groups according to their experience of coronavirus disease 2019 (COVID-19)-related overtime work and LCCOW: (1) “did not experience,” (2) “experienced and was compensated,” and (3) “experienced and was not compensated.” Burnout was measured using the Korean version of the Copenhagen Burnout Inventory, which has 3 subdomains: personal burnout (PB), work-related burnout (WRB), and citizen-related burnout (CRB). Multiple linear regression was applied to examine whether LCCOW was associated with burnout after adjusting for potential confounders.

**RESULTS:**

In total, 74.2% of participants experienced COVID-19-related overtime work, and 14.6% of those who worked overtime experienced LCCOW. COVID-19-related overtime work showed a statistically non-significant association with burnout. However, the association differed by LCCOW. Compared to the “did not experience” group, the “experienced and was not compensated” group was associated with PB (β=10.519; 95% confidence interval [CI], 3.455 to 17.584), WRB (β=10.339; 95% CI, 3.398 to 17.280), and CRB (β=12.290; 95% CI, 6.900 to 17.680), whereas no association was observed for the “experienced and was compensated” group. Furthermore, an analysis restricted to EMS providers who worked overtime due to COVID-19 showed that LCCOW was associated with PB (β=7.970; 95% CI, 1.064 to 14.876), WRB (β=7.276; 95% CI, 0.270 to 14.283), and CRB (β=10.000; 95% CI, 3.435 to 16.565).

**CONCLUSIONS:**

This study suggests that LCCOW could be critical in worsening burnout among EMS providers who worked overtime due to COVID-19.

## INTRODUCTION

Coronavirus disease 2019 (COVID-19), which has caused numerous deaths and suffering worldwide, has also negatively influenced the work environment of healthcare workers [1]. Existing studies have reported that healthcare workers, including medical specialists, nurses, and care workers, are at high risk of being exposed to and contracting COVID-19 [2,3]. Emergency medical services (EMS) providers could be especially vulnerable to COVID-19, given the possibility of prolonged face-to-face contact with patients and increased exposure to infectious aerosols [4].

In response to the COVID-19 pandemic, increased workloads have induced frequent overtime work among healthcare workers [5-8]. For example, research in China found that 54% of healthcare workers experienced overtime work during the COVID-19 pandemic [9]. A study in Australia found that 52.2% of pharmacists had worked overtime due to COVID-19 [10]. Furthermore, a study in Germany found that healthcare workers who worked in COVID-19-related environments reported more overtime hours than those who did not [3].

A growing body of evidence has found that overtime work could harm workers’ mental health [5,11-14]. For example, a study of Japanese workers found that those with longer overtime work showed a higher risk of anxiety, fatigue, and depression than those who worked overtime 20 hours or fewer per month [15]. Also, a few studies found that working overtime during the COVID-19 pandemic could be related to a higher risk of burnout among healthcare workers [9,16]. Considering that burnout among healthcare workers is associated with work performance [17], COVID-19-related overtime work among EMS providers and related mental health outcomes could present public health concerns [18].

Furthermore, several studies have focused on the role of compensation in the association between overtime work and health [19-24]. Suggested compensation for overtime work includes financial compensation (e.g., extra payment) [19-24] and extra rest time (e.g., time off) [20,22,24]. For example, a study of Swedish physicians reported that unpaid overtime was associated with a high level of stress [23]. A cross-sectional survey of physicians in Spain also found that financial compensation for overtime work was related to a lower risk of burnout [19].

In Korea, EMS providers have been responsible for transporting patients who may have COVID-19 during the COVID-19 pandemic [25]. As part of their COVID-19-related work, EMS providers were required to undertake additional tasks, including wearing personal protective equipment, pre-identifying symptoms related to COVID-19, and disinfecting ambulances before returning to the fire department [26]. These work burdens have increased the hours spent on EMS activities [25], which could result in overtime work.

However, few studies have explored the prevalence of overtime work and its potential relationship to health conditions among EMS providers during the COVID-19 pandemic [19]. Considering that all EMS providers in Korea are public officers and responsible for the health and safety of citizens, it is also necessary to investigate the role of organizational factors in the association between overtime work and mental health problems among EMS providers. Therefore, this study analyzed data from EMS providers in Seoul, Korea to answer the following questions:

(1) Is there an association between COVID-19-related overtime work and burnout among Korean EMS providers? Does the association differ by lack of compensation for COVID-19-related overtime work (LCCOW)?

(2) Is there an association between LCCOW and burnout among Korean EMS providers who worked overtime due to COVID-19?

## MATERIALS AND METHODS

### Study population

**Figure 1.**
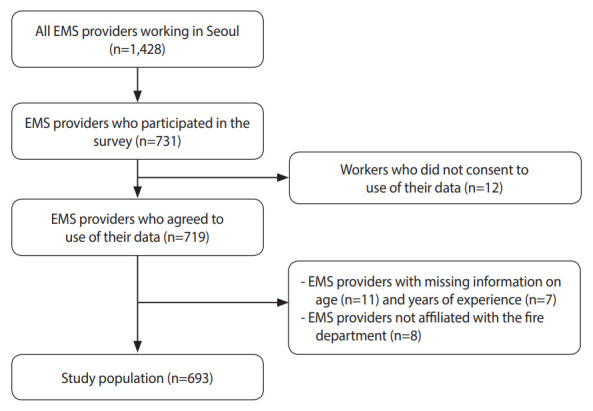
Flow chart of the study population. EMS, emergency medical services.

In 2021, we conducted a cross-sectional survey with the Fire Science Research Center of Seoul Metropolitan Fire Service Academy to investigate the COVID-19-related work environment and health status among EMS providers in Seoul, Korea. Data were collected through an online survey system of Seoul from June 9, 2021 to June 28, 2021. Informed consent was obtained after respondents were provided with an explanation of the research and asked about their willingness to participate. The target population was all EMS providers working in Seoul (n=1,428). Approximately 51% (n=731) of EMS providers participated in the survey. After excluding data from respondents who did not consent to the use of their data (n=12), who have missing information on age (n=11) and years of experience (n=7), and who were not affiliated with the fire department (n=8), the size of the study population was 693 (Figure 1).

### Measures

The experience of COVID-19-related overtime work and LCCOW was measured through the question, “From January 2020 to the present, have you ever worked overtime due to the COVID-19 situation? If yes, have you ever not received adequate compensation (e.g., vacation or additional financial compensation) for the overtime work?” Respondents could answer (1) “did not experience overtime work” (“did not experience”), (2) “experienced overtime work, always received adequate compensation” (“experienced and was compensated”), and (3) “experienced overtime work, did not receive adequate compensation at least once” (“experienced and was not compensated”).

Burnout was measured using the Korean version of the Copenhagen Burnout Inventory (CBI) [27], initially developed by Kristensen et al. [28]. The CBI has 3 domains: personal burnout (PB), work-related burnout (WRB), and client-related burnout. PB indicates the degree of physical and psychological fatigue and exhaustion experienced by a person. WRB indicates the degree of physical and psychological fatigue and exhaustion that is perceived by a person as related to his/her work. Client-related burnout indicates the degree of physical and psychological fatigue and exhaustion that is perceived by a person as related to his/her work with clients. Considering the clients of EMS providers, the concept of client-related burnout was modified to citizen-related burnout (CRB). Each domain consists of 6 items, 7 items, and 6 items, respectively. All items were assessed on a 5-point Likert scale and were scored from 0 to 100 (always/to a very high degree=100, often/to a high degree=75, sometimes/somewhat=50, seldom/to a low degree=25, and never/almost never/to a very low degree=0). The fourth item for WRB was reverse-coded because it measured a positive concept. The average score was calculated for each of the 3 domains, and the scoring range was 0 to 100 per domain. Higher scores indicated a higher degree of burnout. Cronbach’s alpha was 0.938 for PB, 0.913 for WRB, and 0.925 for CRB.

As potential confounders, 5 variables were selected: age, sex, household size, job rank, and years of experience. Age was classified into 4 categories (21-30, 31-35, 36-40, and 41-60 years old). All respondents were categorized as male or female. Household size was grouped into 4 categories (1, 2, 3, or ≥ 4 people). Job rank was classified into 4 categories (*sobang-sa, sobang-gyo, sobang-jang, sobang-wi* or higher). Years of experience was coded into 4 categories (< 5, 5-9, 10-14, ≥ 15 years).

Additionally, COVID-19-related workload variables were measured for the period from January 2020 until the survey. Yes/no questions were used to measure several experiences: receiving COVID-19 screening test, COVID-19-related self-quarantine, COVID-19 infection, and not going home after work because of fear of transmitting COVID-19 to the family. A perceived increase in workload after the outbreak of COVID-19 compared to 2019 was also measured by a yes/no question. The experience of lack of time for administrative work due to an increase in fieldwork, difficulty in selecting a hospital to transfer a patient, transferring the patient to the outside of service area, and waiting more than an hour after transferring the patient to the hospital were measured and classified into 2 categories: no (“no” and “not applicable”) and yes (yes).

### Statistical analysis

A multiple linear regression model was applied to investigate the role of LCCOW in the association between COVID-19-related overtime work and burnout among EMS providers, after adjusting for confounders. Since burnout and COVID-19-related overtime work among EMS providers from the same fire department could be correlated, cluster-robust standard errors were applied using information about participants’ affiliated fire departments. All confounders were included as categorical variables in the analysis. Results were presented as coefficients with 95% confidence intervals (CIs). All statistical analyses were performed with Stata/SE version 17.0 (Stata Corp., College Station, TX, USA).

### Ethics statement

This study was approved by the Institutional Review Board of Korea University (KUIRB-2021-0163-01).

## RESULTS

Table 1 shows the distribution of the study population and the experience of COVID-19-related overtime work by each covariate. Overall, 74.2% (n=514) of the study population reported that they experienced COVID-19-related overtime work. Experience of COVID-19-related overtime work was prevalent among EMS providers who were aged 31-35 years old, had fewer years of experience, and had a lower job rank. Also, the prevalence of COVID-19-related overtime work was higher among EMS providers with COVID-19-related workloads, including the experience of difficulty of selecting a hospital to transfer a patient (Supplementary Material 1). The prevalence of LCCOW among those who worked overtime did not differ by the characteristics of the study population, including COVID-19-related workload (Supplementary Material 2). The average burnout score was higher among EMS providers who were female, lived in a 2-person household, had 5-9 years of experience, held the *sobang-gyo* rank, and had a COVID-19-related workload (Supplementary Material 3).

Experience of COVID-19-related overtime work showed a statistically non-significant association with burnout (Table 2). However, after being stratified by LCCOW, statistically significantly higher scores of PB (β=10.519; 95% CI, 3.455 to 17.584), WRB (β=10.339; 95% CI, 3.398 to 17.280), and CRB (β=12.290; 95% CI, 6.900 to 17.680) were observed among the “experienced and was not compensated” group when compared to the “did not experience” group, though not among the “experienced and was compensated” group (Table 2).

We also conducted an analysis restricted to EMS providers who had worked overtime due to COVID-19 (Table 3). LCCOW was significantly associated with burnout among EMS providers after adjusting for confounders, including COVID-19-related workload. Compared to the “experienced and was compensated” group, the “experienced and was not compensated” group showed higher scores for PB (β=7.970; 95% CI, 1.064 to 14.876), WRB (β=7.276; 95% CI, 0.270 to 14.283), and CRB (β=10.000; 95% CI, 3.435 to 16.565).

## DISCUSSION

This study found that approximately 75% of EMS providers in Seoul experienced COVID-19-related overtime work from January 2020 to June 2021, and about 15% of EMS providers who worked overtime experienced LCCOW. The burden of overtime work during the COVID-19 pandemic could constitute a public health concern, considering potential long-term effects on the health of EMS providers [29]. This study also showed that the average burnout score of EMS providers in Seoul was above 50, which could indicate a high level of burnout [27]. A high level of burnout among EMS providers is a public health concern because it could lead to turnover intention and decreased work performance, which would increase the burden on the healthcare system [30,31].

Our findings suggest that overtime was not associated with burnout among EMS providers, which is inconsistent with previous studies [32,33]. Notably, however, we found that compensation might play a critical role in the association between COVID-19-related overtime work and burnout among EMS providers, which is consistent with a study of German workers [34]. A statistically significantly higher degree of burnout was observed among EMS providers who did not receive adequate compensation for COVID-19-related overtime work than among those who did not work overtime. We also found that LCCOW was related to burnout among EMS providers who worked overtime even after adjusting for COVID-19-related workload.

There could be several explanations for our results, considering the examples of compensation for COVID-19-related overtime work indicated in our questionnaire (e.g., vacation, additional financial compensation). First, it is possible that the EMS providers who worked overtime did not receive enough time to recover from fatigue. A study of German workers found that boundaryless working hours, including overtime, could negatively influence workers’ states of recovery [35]. Second, an imbalance between financial/non-financial compensation and COVID-19-related overtime work might lead to higher burnout among EMS providers. Previous studies reported that a lack of rewards for workers’ efforts could lead to emotional distress [36] and work dissatisfaction [24]. Therefore, LCCOW might imply an imbalance between EMS providers’ efforts, including overtime work, and rewards from the organization, including vacation, additional financial compensation, and promotion, which could increase the risk of burnout among EMS providers [37-39].

We also found a statistically non-significant difference in burnout scores between EMS providers who always received adequate compensation for COVID-19-related overtime work and those who did not work overtime. This result suggests that organizational rewards could reduce burnout among EMS providers who were required to engage in overtime work during the COVID-19 pandemic. However, we should interpret these results cautiously, in that EMS providers who did not work overtime also reported higher burnout than the levels observed among Korean workers before the COVID-19 pandemic [27]. Therefore, it is also necessary to explore the organizational factors (e.g., staffing adequacy) that could reduce burnout among Korean EMS providers.

This study has several limitations. First, information on the temporal order between COVID-19-related overtime work and burnout could not be provided due to the cross-sectional design of the survey. Therefore, future longitudinal studies should adjust for potential confounders, including the degree of burnout at baseline. Second, there could be healthy worker survival effects. For example, EMS providers with severe burnout might have taken a leave of absence or resigned from their jobs, such that they did not participate in the survey. Excessive overtime work might also have prevented EMS providers from participating in the survey. Third, because a single question was used to assess COVID-19-related overtime work, the duration of overtime work could not be considered. Future studies should control for overtime work hours when examining the association between LCCOW and burnout. Fourth, the nature and magnitude of compensation for COVID-19-related overtime work were not measured. Future studies might investigate which types and what extent of compensation could buffer the impact of COVID-19-related overtime work on burnout among EMS providers, including the total hours of overtime work compensated and satisfaction with the corresponding amount of compensation.

Using data from EMS providers in Seoul, this study showed that the association between overtime during the COVID-19 pandemic and burnout among EMS providers differed by LCCOW, and EMS providers who did not receive adequate compensation for overtime work had the highest burnout scores. LCCOW was also associated with burnout among EMS providers who worked overtime due to COVID-19. These findings imply that LCCOW could play a critical role in worsening burnout among EMS providers who worked overtime during the COVID-19 pandemic.

## Figures and Tables

**Table 1.** Distribution of study population and COVID-19-related overtime work by key covariates among EMS providers in Seoul

Variables		Total	Experience of COVID-19-related overtime work	p-value^1^
Overall		693 (100)	514 (74.2)	
Sex				0.123
	Male	572 (82.5)	431 (75.3)	
	Female	121 (17.5)	83 (68.6)	
Age (yr)				<0.001
	21-30	147 (21.2)	110 (74.8)	
	31-35	245 (35.4)	206 (84.1)	
	36-40	171 (24.7)	111 (64.9)	
	41-60	130 (18.8)	87 (66.9)	
Household size				0.244
	One person	133 (19.2)	106 (79.7)	
	Two people	155 (22.4)	118 (76.1)	
	Three people	178 (25.7)	130 (73.0)	
	Four people or more	227 (32.8)	160 (70.5)	
Years of experience (yr)				<0.001
	<5	266 (38.4)	208 (78.2)	
	5-9	219 (31.6)	173 (79.0)	
	10-14	111 (16.0)	76 (68.5)	
	≥15	97 (14.0)	57 (58.8)	
Job rank				0.002
	*Sobang-sa* ^2^	217 (31.3)	174 (80.2)	
	*Sobang-gyo*	287 (41.4)	219 (76.3)	
	*Sobang-jang*	140 (20.2)	91 (65.0)	
	*Sobang-wi* or higher	49 (7.1)	30 (61.2)	

Values are presented as number (%). COVID-19, coronavirus disease 2019; EMS, emergency medical services.

1 p‐value of the chi‐square test comparing the prevalence of overtime work across different groups.

2 Lowest.

**Table 2.** The role of LCCOW in the association between COVID-19-related overtime work and burnout among EMS providers in Seoul (n=693)^1^

COVID-19-related overtime work		Total	PB	WRB	CRB
Did not experience		179 (25.8)	Reference	Reference	Reference
Experienced		514 (74.2)	2.495 (-2.332, 7.322)	2.983 (-1.351, 7.316)	3.128 (-0.079, 6.336)
Stratified by LCCOW					
	Did not experience	179 (25.8)	Reference	Reference	Reference
	Experienced and was compensated	439 (63.4)	1.085 (-3.703, 5.872)	1.690 (-2.540, 5.920)	1.518 (-1.962, 4.999)
	Experienced and was not compensated	75 (10.8)	10.519 (3.455, 17.584)^**^	10.339 (3.398, 17.280)^**^	12.290 (6.900, 17.680)^***^

Values are presented as number (%) or β (95% confidence interval). LCCOW, lack of compensation for COVID-19-related overtime work; COVID-19, coronavirus disease 2019; EMS, emergency medical services; PB, personal burnout; WRB, work-related burnout; CRB, citizen-related burnout.

1 Adjusted for age, sex, household size, job rank, and years of experience.

** p<0.01,

*** p<0.001.

**Table 3.** Associations between LCCOW and burnout among EMS providers in Seoul who worked overtime (n=514)^1^

LCCOW	Total	PB	WRB	CRB
Compensated	439 (85.4)	Reference	Reference	Reference
Not compensated	75 (14.6)	7.970 (1.064, 14.876)^*^	7.276 (0.270, 14.283)^*^	10.000 (3.435, 16.565)^**^

Values are presented as number (%) or β (95% confidence interval). LCCOW, lack of compensation for COVID-19-related overtime work; COVID-19, coronavirus disease 2019; EMS, emergency medical services; PB, personal burnout; WRB, work-related burnout; CRB, citizen-related burnout.

1 Adjusted for age, sex, household size, job rank, years of experience, and COVID-19-related workload.

* p<0.05,

** p<0.01.

## SUPPLEMENTARY MATERIALS

Supplement Material 1. Distribution of study population and COVID-19-related overtime work by COVID-19-related workloads among EMS providers in Seoul (N=693)

Supplement Material 2. Distribution of study population and LCCOW by key covariates among EMS providers who worked overtime (N=514)

Supplement Material 3. Distribution of burnout by key covariates among EMS providers in Seoul (N=693)
