# Supplementary material for: Lack of compensation for COVID-19-related overtime work and its association with burnout among EMS providers in Korea
Source: Epidemiol Health. 2023 Jun 15;45:e2023058. doi: 10.4178/epih.e2023058 (PMC10667576; doi:10.4178/epih.e2023058)
Supplement: Supplement Material 1. — Distribution of study population and COVID-19-related overtime work by COVID-19-related workloads among EMS providers in Seoul (N=693) [file epih-45-e2023058-Supplementary-1.docx]

Supplementary Material 1. Distribution of study population and COVID-19-related overtime work by COVID-19-related workloads among EMS providers in Seoul (N=693)

|  | Total | Experience of  COVID-19-related  overtime work | p-value^1^ |
| --- | --- | --- | --- |
|  | N (%) | N (%) |  |
| Overall | 693 (100.0) | 514 (74.2) |  |
| Received COVID-19 Screening test |  |  | <0.001 |
| No | 382 (55.1) | 257 (67.3) |  |
| Yes | 311 (44.9) | 257 (82.6) |  |
| COVID-19-related self-quarantine |  |  | <0.001 |
| No | 349 (50.4) | 234 (67.0) |  |
| Yes | 344 (49.6) | 280 (81.4) |  |
| Experience of COVID-19 infection |  |  | 0.960 |
| No | 685 (98.8) | 508 (74.2) |  |
| Yes | 8 (1.2) | 6 (75.0) |  |
| Experience of not going home after work |  |  | <0.001 |
| No | 516 (74.5) | 365 (70.7) |  |
| Yes | 177 (25.5) | 149 (84.2) |  |
| Perceived increase in workload |  |  | 0.009 |
| No | 48 (6.9) | 28 (58.3) |  |
| Yes | 645 (93.1) | 486 (75.3) |  |
| Experience of lack of time for  administrative work |  |  | 0.017 |
| No | 53 (7.6) | 32 (60.4) |  |
| Yes | 640 (92.4) | 482 (75.3) |  |
| Experience of difficulty in  selecting a hospital to transfer a patient |  |  | <0.001 |
| No | 27 (3.9) | 11 (40.7) |  |
| Yes | 666 (96.1) | 503 (75.5) |  |
| Experience of transferring the patient  to the outside of service area |  |  | <0.001 |
| No | 34 (4.9) | 16 (47.1) |  |
| Yes | 659 (95.1) | 498 (75.6) |  |
| Experience of waiting more than an hour  after transferring the patient to the hospital |  |  | <0.001 |
| No | 24 (3.5) | 10 (41.7) |  |
| Yes | 669 (96.5) | 504 (75.3) |  |
| EMS, emergency medical services; COVID-19, coronavirus disease 2019.  ^1^P‐value of the chi‐square test comparing the prevalence of overtime work across different groups. | | | |
