# Supplementary material for: Lack of compensation for COVID-19-related overtime work and its association with burnout among EMS providers in Korea
Source: Epidemiol Health. 2023 Jun 15;45:e2023058. doi: 10.4178/epih.e2023058 (PMC10667576; doi:10.4178/epih.e2023058)
Supplement: Supplement Material 2. — Distribution of study population and LCCOW by key covariates among EMS providers who worked overtime (N=514) [file epih-45-e2023058-Supplementary-2.docx]

Supplementary Material 2. Distribution of study population and LCCOW by key covariates among EMS providers who worked overtime (N=514)

|  | Total | LCCOW | p-value^1^ |
| --- | --- | --- | --- |
|  | N (%) | N (%) |  |
| Overall | 514 (100.0) | 75 (14.6) |  |
| Sex |  |  | 0.706 |
| Male | 431 (83.9) | 64 (14.8) |  |
| Female | 83 (16.1) | 11 (13.3) |  |
| Age (yr) |  |  | 0.503 |
| 21~30 | 110 (21.4) | 14 (12.7) |  |
| 31~35 | 206 (40.1) | 27 (13.1) |  |
| 36~40 | 111 (21.6) | 21 (18.9) |  |
| 41~60 | 87 (16.9) | 13 (14.9) |  |
| Household size |  |  | 0.236 |
| One person | 106 (20.6) | 20 (18.9) |  |
| Two people | 118 (23.0) | 12 (10.2) |  |
| Three people | 130 (25.3) | 22 (16.9) |  |
| Four people or more | 160 (31.1) | 21 (13.1) |  |
| Years of experience (yr) |  |  | 0.435 |
| <5 | 208 (40.5) | 26 (12.5) |  |
| 5~9 | 173 (33.7) | 27 (15.6) |  |
| 10~14 | 76 (14.8) | 15 (19.7) |  |
| ≥15 | 57 (11.1) | 7 (12.3) |  |
| Job rank |  |  | 0.605 |
| *Sobang-sa*^2^ (lowest) | 174 (33.9) | 22 (12.6) |  |
| *Sobang-gyo* | 219 (42.6) | 34 (15.5) |  |
| *Sobang-jang* | 91 (17.7) | 16 (17.6) |  |
| *Sobang-wi or higher* | 30 (5.8) | 3 (10.0) |  |
| Received COVID-19 Screening test |  |  | 0.382 |
| No | 257 (50.0) | 34 (13.2) |  |
| Yes | 257 (50.0) | 41 (16.0) |  |
| COVID-19-related self-quarantine |  |  | 0.474 |
| No | 234 (45.5) | 37 (15.8) |  |
| Yes | 280 (54.5) | 38 (13.6) |  |
| Experience of COVID-19 infection |  |  | 0.308 |
| No | 508 (98.8) | 75 (14.8) |  |
| Yes | 6 (1.2) | 0 (0.0) |  |
| Experience of not going home after work |  |  | 0.534 |
| No | 365 (71.0) | 51 (14.0) |  |
| Yes | 149 (29.0) | 24 (16.1) |  |
| Perceived increase in workload |  |  | 0.251 |
| No | 28 (5.4) | 2 (7.1) |  |
| Yes | 486 (94.6) | 73 (15.0) |  |
| Experience of lack of time for administrative work |  |  | 0.729 |
| No | 32 (6.2) | 4 (12.5) |  |
| Yes | 482 (93.8) | 71 (14.7) |  |
| Experience of difficulty in selecting a hospital to transfer a patient |  |  | 0.601 |
| No | 11 (2.1) | 1 (9.1) |  |
| Yes | 503 (97.9) | 74 (14.7) |  |
| Experience of transferring the patient to the outside of service area |  |  | 0.337 |
| No | 16 (3.1) | 1 (6.3) |  |
| Yes | 498 (96.9) | 74 (14.9) |  |
| Experience of waiting more than an hour after transferring the patient to the hospital |  |  | 0.678 |
| No | 10 (1.9) | 1 (10.0) |  |
| Yes | 504 (98.1) | 74 (14.7) |  |
| EMS, emergency medical services; COVID-19, coronavirus disease 2019; LCCOW, lack of compensation for COVID-19-related overtime work ^1^P‐value of the chi‐square test comparing the prevalence of lack of compensation for COVID-19-related overtime work across different groups.  ^2^Lowest. | | | |
