# Supplementary material for: Lack of compensation for COVID-19-related overtime work and its association with burnout among EMS providers in Korea
Source: Epidemiol Health. 2023 Jun 15;45:e2023058. doi: 10.4178/epih.e2023058 (PMC10667576; doi:10.4178/epih.e2023058)
Supplement: Supplement Material 3. — Distribution of burnout by key covariates among EMS providers in Seoul (N=693) [file epih-45-e2023058-Supplementary-3.docx]

Supplementary Material 3. Distribution of burnout by key covariates among EMS providers in Seoul (N=693)

|  | PB | WRB | CRB |
| --- | --- | --- | --- |
|  | Mean (SD) | Mean (SD) | Mean (SD) |
| Overall | 54.4 (24.0) | 53.9 (22.8) | 53.7 (24.6) |
| Sex |  |  |  |
| Male | 53.3 (24.4) | 52.8 (23.3) | 52.5 (25.0) |
| Female | 59.5 (21.3) | 58.9 (19.4) | 59.3 (21.7) |
| Age (yr) |  |  |  |
| 21~30 | 54.5 (22.1) | 55.9 (21.2) | 53.7 (21.8) |
| 31~35 | 55.9 (23.4) | 55.6 (22.7) | 55.1 (26.0) |
| 36~40 | 56.5 (25.0) | 55.6 (23.4) | 55.4 (24.7) |
| 41~60 | 48.4 (25.1) | 46.1 (22.5) | 48.8 (24.4) |
| Household size |  |  |  |
| One person | 55.9 (24.3) | 55.2 (23.7) | 53.8 (24.4) |
| Two people | 58.3 (23.1) | 58.5 (21.2) | 57.4 (23.5) |
| Three people | 54.7 (23.1) | 54.1 (22.6) | 53.6 (24.8) |
| Four people or more | 50.5 (24.8) | 49.8 (22.9) | 51.2 (25.1) |
| Years of experience (yr) |  |  |  |
| <5 | 53.9 (22.4) | 53.8 (22.2) | 50.4 (22.9) |
| 5~9 | 57.7 (23.0) | 57.4 (22.0) | 57.8 (24.5) |
| 10~14 | 54.9 (28.1) | 53.7 (25.3) | 57.3 (27.6) |
| ≥15 | 47.5 (24.1) | 46.3 (21.4) | 49.4 (23.9) |
| Job rank |  |  |  |
| *Sobang-sa*^1^ | 52.2 (21.9) | 52.2 (21.8) | 50.0 (22.7) |
| *Sobang-gyo* | 57.7 (23.4) | 57.4 (22.1) | 56.3 (24.2) |
| *Sobang-jang* | 52.8 (27.3) | 51.8 (24.6) | 56.2 (27.3) |
| *Sobang-wi or higher* | 48.6 (24.3) | 46.6 (22.3) | 47.5 (24.4) |
| Received COVID-19 Screening test |  |  |  |
| No | 52.7 (23.4) | 52.3 (22.3) | 53.5 (24.4) |
| Yes | 56.4 (24.7) | 55.8 (23.3) | 54.0 (24.9) |
| COVID-19-related self-quarantine |  |  |  |
| No | 54.3 (23.0) | 53.1 (21.8) | 53.7 (24.4) |
| Yes | 54.4 (25.0) | 54.7 (23.7) | 53.7 (24.8) |
| Experience of COVID-19 infection |  |  |  |
| No | 54.3 (23.9) | 53.8 (22.7) | 53.7 (24.5) |
| Yes | 59.9 (32.1) | 58.5 (32.0) | 54.7 (33.7) |
| Experience of not going home after work |  |  |  |
| No | 51.1 (23.6) | 50.7 (22.1) | 51.2 (24.2) |
| Yes | 63.9 (22.7) | 63.1 (22.1) | 60.9 (24.4) |
| Perceived increase in workload |  |  |  |
| No | 37.8 (17.5) | 34.4 (15.7) | 36.2 (19.9) |
| Yes | 55.6 (24.0) | 55.3 (22.6) | 55.0 (24.4) |
| Experience of lack of time for administrative work |  |  |  |
| No | 34.2 (21.8) | 32.7 (19.2) | 36.6 (23.9) |
| Yes | 56.0 (23.4) | 55.6 (22.2) | 55.1 (24.1) |
| Experience of difficulty in selecting a hospital to transfer a patient |  |  |  |
| No | 42.9 (25.6) | 41.7 (23.7) | 45.5 (26.9) |
| Yes | 54.8 (23.8) | 54.4 (22.6) | 54.0 (24.5) |
| Experience of transferring the patient to the outside of service area |  |  |  |
| No | 39.3 (23.3) | 38.2 (20.6) | 43.5 (26.3) |
| Yes | 55.1 (23.8) | 54.7 (22.6) | 54.2 (24.4) |
| Experience of waiting more than an hour after transferring the patient to the hospital |  |  |  |
| No | 38.7 (23.8) | 37.5 (19.9) | 39.9 (26.0) |
| Yes | 54.9 (23.8) | 54.5 (22.7) | 54.2 (24.4) |
| EMS, emergency medical services; COVID-19, coronavirus disease 2019; PB, Personal burnout; WRB, Work-related burnout; CRB, Citizen-related burnout.  ^1^Lowest | | | |
